# The Deep Fascia of the Forearm and the Ulnar Nerve: An Anatomical Study

**DOI:** 10.7759/cureus.2842

**Published:** 2018-06-19

**Authors:** Paul J Choi, Chidinma Nwaogbe, Joe Iwanaga, Georgi P Georgiev, Rod J Oskouian, R. Shane Tubbs

**Affiliations:** 1 Surgery, Seattle Science Foundation, Seattle, USA; 2 Molecular, Cellular & Biomedical Sciences, CUNY School of Medicine, New York, USA; 3 Seattle Science Foundation, Seattle, USA; 4 Orthopaedics and Traumatology, University Hospital Queen Giovanna, Sofia, BGR; 5 Neurosurgery, Swedish Neuroscience Institute, Seattle, USA; 6 Neurosurgery, Seattle Science Foundation, Seattle, USA

**Keywords:** recurrent cubital tunnel syndrome, flexor carpi ulnaris, ulnar nerve, nerve entrapment, revision surgery

## Abstract

Introduction

A reoperation for a cubital tunnel syndrome is not uncommon. Patients often complain of sensorimotor symptoms in the ulnar nerve distribution after their primary surgery. The documented etiologies for such a phenomenon include a “new” kinking of the distal ulnar nerve and a “new” compression of the ulnar nerve by the fascial septum in between or tendinous bands over the muscles of the forearm. The deep fascial plane along which the ulnar nerve travels in the forearm has had scant attention. We present an anatomical study to provide a better understanding of such etiologies to aid physicians in performing successful primary ulnar nerve release that does not lead to risky reoperations and ultimately yields improved patient satisfaction.

Materials and methods

The forearms of 12 fresh frozen cadavers (24 arms) underwent dissection, during which the fascial relationships between the ulnar nerve and muscles of the anterior compartment were explored with a blunt technique. The relationship between the fascial planes and the ulnar nerve was quantitatively and qualitatively documented. The ranges of motion of the elbow were also observed for any potential compression points on the nerve during the movement.

Results

In all specimens (n = 24), the ulnar nerve entered the forearm between the humeral and ulnar heads of the flexor carpi ulnaris, after which it routed deep to a deep fascia between the anterior surface of the flexor carpi ulnaris and the posterior surface of the flexor digitorum superficialis. Ulnar nerve branches to the flexor carpi ulnaris pierced this fascial septum while en route to the posterior surface of the muscle. Medially, the branches to the flexor digitorum profundus also pierced this fascial plane. In most arms, the fascia became thinner near the junction between the proximal two-thirds and distal one-third of the forearm. On no side was the ulnar nerve found to be grossly compressed by this deep fascia. However, with the extension of the elbow, a degree of angulation of the proximal ulnar nerve was observed due to its compact connection with the deep fascia.

Conclusion

Our study revealed that there is an intimate relationship between the ulnar nerve and the deep fascia of the forearm. Since the ulnar branches to the overlying flexor carpi ulnaris pierce this deep structure, a care should be given to its anatomical course during surgery in this region to prevent denervation of the muscle.

## Introduction

Reoperation for patients with cubital tunnel syndrome is not uncommon. Mackinnon and Novak retrospectively reviewed 100 patients who had undergone a second surgery for their previous cubital tunnel syndrome [1]. Of these subjects, a distal kink of the ulnar nerve was found in 57 patients [1]. Etiologies for such distal kinks include the formation of “tendinous bands” at the flexor/pronator muscle origin. These authors found that a distal ulnar nerve compression by the medial intermuscular septum was twice as common as a proximal compression by the septum [1, 2]. Filippi et al. reported 22 patients who underwent reoperation of the ulnar nerve at the elbow and found that the symptoms were due to perineural fibrosis and scarring, adhesion of the ulnar nerve to the medial epicondyle, and incomplete excision of the medial intermuscular septum, which are the causes of 90% of all reoperations [1-4]. Mackinnon and Novak have stated that primary surgery for a cubital tunnel syndrome with anterior transposition can create new, iatrogenic compressive sites, especially distally, if the nerve is not fully released. They recommend a complete excision of the distal septum located between the flexor carpi ulnaris and flexor/pronator origin to ensure that the ulnar nerve can be transposed anteriorly without becoming kinked or compressed [1, 2].

## Materials and methods

The forearms of 12 fresh frozen cadavers (24 arms) underwent dissection. Eight specimens were female and four were male with a mean age of 84.5 years at death. Specifically, the fascial relationships between the ulnar nerve and muscles of the anterior compartment were studied with blunt dissection. When necessary, loupe magnification was used. Observations of the fascial relationships of the ulnar nerve in the forearm were recorded. Ranges of motion of the elbow were also studied to note any compression of the ulnar nerve by the deep fascia of the forearm.

## Results

**Figure 1:**
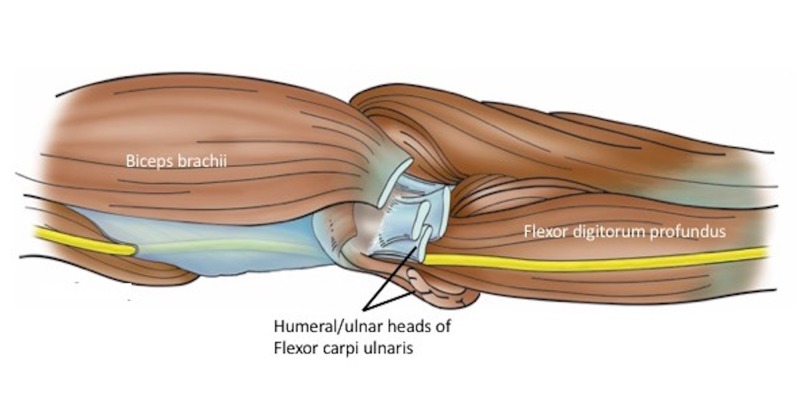
Schematic drawing of the ulnar nerve (yellow) in the distal left arm and proximal forearm and its muscular relationships.

**Figure 2:**
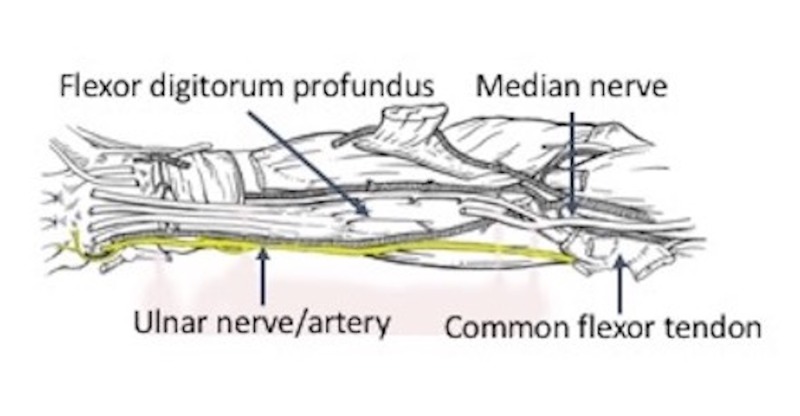
Schematic drawing of regional neurovascular structures related to the ulnar nerve (yellow) in the right forearm.

**Figure 3:**
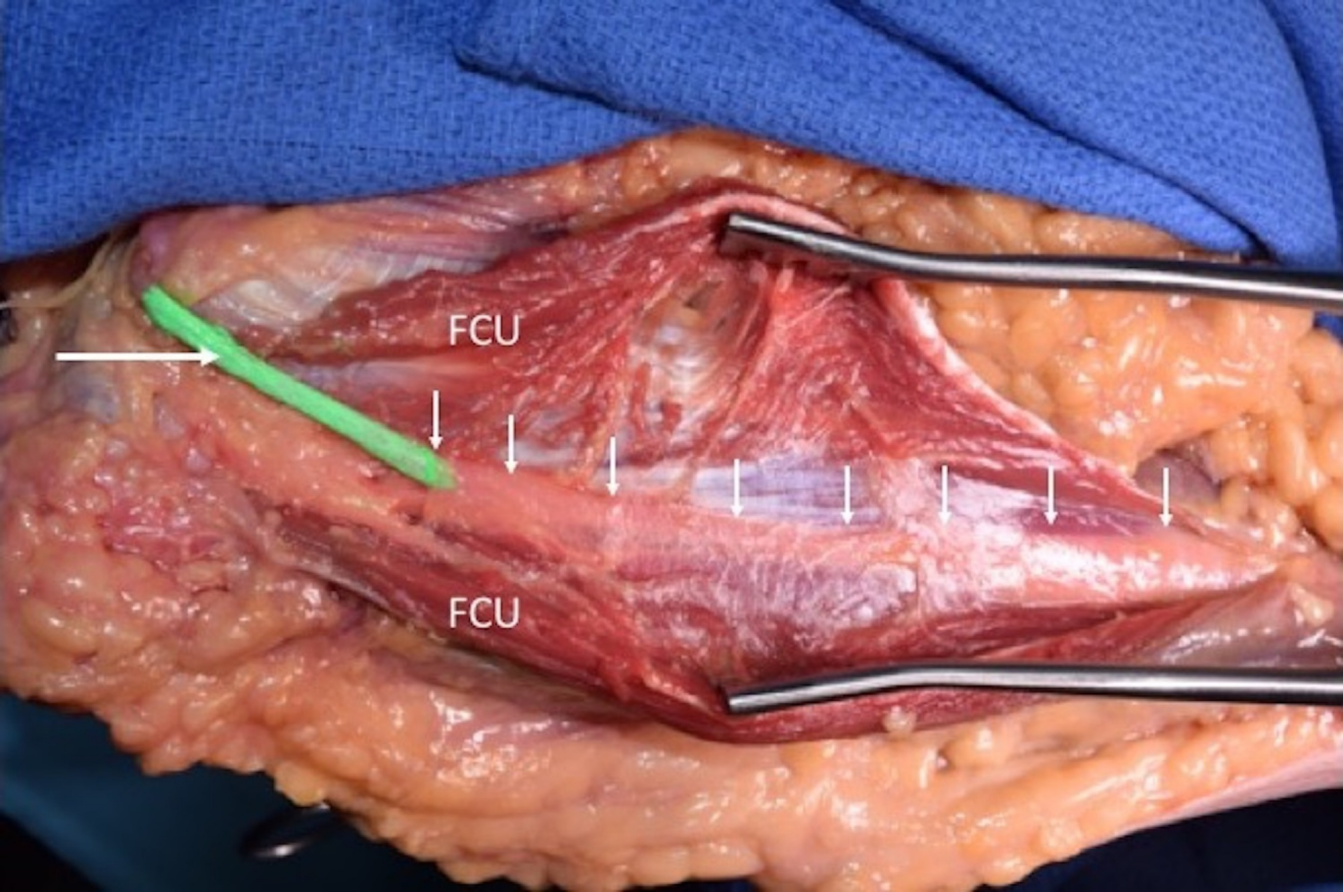
Left cadaveric specimen demonstrating the ulnar nerve (green with arrow on part behind medial epicondyle). The flexor carpi ulnaris (FCU) is opened with a retractor to reveal the nerve (uncolored) as it courses deep (arrows) to the deep fascia of the forearm.

**Figure 4:**
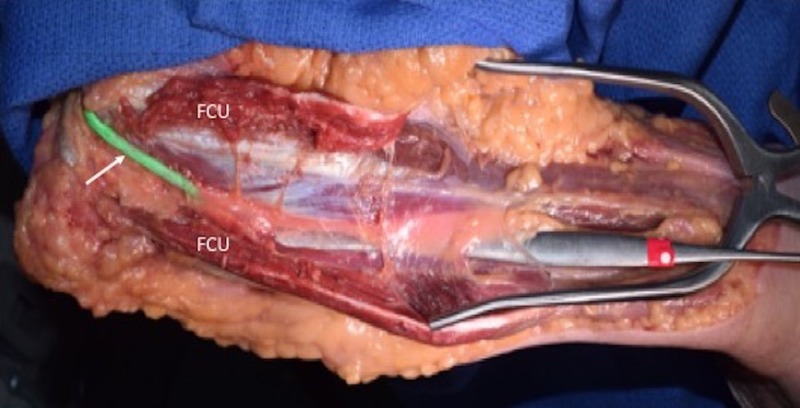
A dissector is placed under deep fascia of the forearm i.e., in the space where the ulnar nerve travels. FCU: Flexor carpi ulnaris.

**Figure 5:**
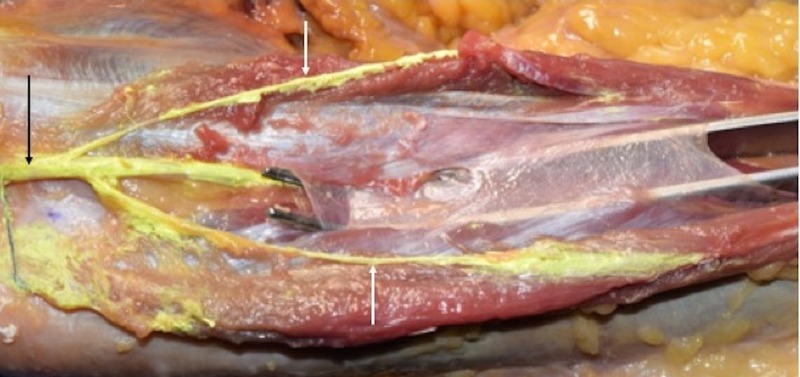
Left cadaveric forearm noting the proximal ulnar nerve (black arrow) and its branches (white arrows) to the flexor carpi ulnaris. Notice that these branches are outside the deep fascia of the forearm but that the main ulnar nerve trunk continues deep to the fascia in the space elevated by the forceps.

In all specimens, the ulnar nerve entered the forearm between the humeral origin (on the medial epicondyle) and ulnar origin of the flexor carpi ulnaris muscle (Figures 1, 2). The nerve then traveled into the deep fascia between the anterior surface of the flexor carpi ulnaris and the posterior surface of the flexor digitorum superficialis muscles (Figures 3, 4). The ulnar branches to the flexor carpi ulnaris arose proximal to the septum and branches to the flexor digitorum profundus pierced this fascial septum while in route to the posterior surface of this muscle’s ulnar one half (Figure 5). Medially, the ulnar branches to the flexor digitorum profundus also pierced this fascial plane. In most arms, the fascia became thinner near the junction between the proximal two-thirds and distal one-third of the forearm. The mean length, width, and thickness of the septum were 17.5 cm (a range of 16-25 cm), 2.5 cm (a range of 2-3.8 cm), and 0.35 mm (a range of 0.3-0.45 mm), respectively. In approximately 20% of the arms, the proximal thicker part of this deep fascia was seen as horizontally traveling white bands. Upon inspection, this deep fascia extended medially toward the ulnar artery and laterally toward the radius. On no side was the ulnar nerve found to be grossly compressed by this deep fascia. However, with the extension of the elbow, some angulation of the proximal ulnar nerve was noted due to its intimate connection to the deep fascia.

## Discussion

Mackinnon and Novak conclude that a greater awareness of the potential for distal and new entrapment sites would significantly improve patient outcome following a cubital tunnel release procedure [1, 2]. As can be seen in our study, an intimate relationship of the ulnar nerve and the deep fascia exists, which may play a role in distorting the angle of the nerve upon extension of the forearm. The deep fascia is an anatomically tough structure that lies immediately against the course of the ulnar nerve. This anatomical correlation explains the significant reoperation rate, i.e., 10 out of 14 patients in the report by Filippi et al. and >90% in the report by Tang et al. after an ulnar nerve release operation [2, 5]. This is attributable to an incomplete resection of the deep fascia during the primary operation and postoperative scar tissue formation, leading to iatrogenic compressions of the ulnar nerve and subsequent recurrent neuropathy.

Campbell et al. conducted a review on 100 cadaveric specimens after a patient with ulnar neuropathy demonstrated focal conduction block at the point where the ulnar nerve exited the flexor carpi ulnaris in the forearm [6]. While dissecting, it was found that the intermuscular septum between the flexor carpi ulnaris and flexor digitorum profundus muscles was thickened in several specimens, indicating that the septum may represent a site of ulnar nerve entrapment. A study conducted by Amadio and Beckenbaugh found that the deep flexor-pronator aponeurosis between the humeral and ulnar heads of the flexor carpi ulnaris may be a potential site for constriction of the ulnar nerve [7]. It was reported in two cases of compression at this structure, decompression alleviated symptoms of ulnar nerve entrapment. Green and Rayan reported similar findings to Amadio and Beckenbaugh and reported evidence of increased pressures on the ulnar nerve at the common aponeurosis, independent of transposition or proximal decompression [8]. Inserra and Spinner identified the common aponeurosis of the flexor digitorum superficialis and flexor carpi ulnaris as a tether of the ulnar nerve on attempted transposition of the nerve [9]. Gonzalez et al. also described a similar fibrous tunnel in 17 of the 39 specimens formed by the flexor pronator aponeurosis [10]. Karatas et al. also reported fibrous thickening between the flexor digitorum superficialis and flexor carpi ulnaris on five sides [11]. Won et al. found that segments of the intermuscular aponeurosis between the flexor digitorum superficialis and the flexor carpi ulnaris as well as the intermuscular aponeurosis between the flexor digitorum profundus and flexor carpi ulnaris are also potential causes of ulnar nerve compression [12]. Overall, these four reports had a common finding of a fibrous aponeurosis between the flexor digitorum superficialis and the humeral head of the flexor carpi ulnaris through which the ulnar nerve traversed [9].

Ahcan and Zorman suggest that endoscopic nerve release for cubital tunnel syndrome retains all the benefits of an open surgery while reducing the rate of scar tissue formation. They were able to achieve an ideal surgical field and perform complete resection of potential compression sites endoscopically [13]. Significant degrees of improvement were noted in all of the 36 patients who were treated endoscopically and 64% had a complete recovery without scarring or contracture seen during their 16-month follow-up and only one patient had a recurrence [13]. On the other hand, Tang et al. suggest that the endoscopic technique carries an equal success rate as do the other techniques, e.g., medial epicondylectomy, anterior transposition, in situ decompression [1, 2, 5].

A revision surgery for cubital tunnel syndrome carries a high risk of ulnar nerve damage since the dense fibrosis and scar tissue, which develop after the primary operation, prevents a safe dissection of the nerve [5]. Therefore, it is best to perform a successful one-time procedure, which does not lead to a risky reoperation.

## Conclusions

We identified a close structural relationship of the ulnar nerve and the deep fascia of the anterior compartment of the forearm via cadaveric dissections. This relationship explains the recurrent nature and the significant reoperation rate of the cubital tunnel syndrome. It is advisable that the physician performing the nerve release operation understands this structural anatomy and that a complete longitudinal dissection of the deep fascia is required to minimize reoperation rate and achieve improved patient satisfaction. Moreover, an endoscopic approach is becoming more popular for utilization in the management of cubital tunnel syndrome.
